# Involvement of Oxidative Stress in Mitochondrial Abnormalities During the Development of Heart Disease

**DOI:** 10.3390/biomedicines13061338

**Published:** 2025-05-29

**Authors:** Naranjan S. Dhalla, Petr Ostadal, Paramjit S. Tappia

**Affiliations:** 1 St. Boniface Hospital Albrechtsen Research Centre, Institute of Cardiovascular Sciences, Department of Physiology & Pathophysiology, Max Rady College of Medicine, University of Manitoba, Winnipeg, MB R2H 2A6, Canada; 2 Department of Cardiology, 2nd Faculty of Medicine, Charles University, Motol University Hospital, V Uvalu 84, 15000 Prague, Czech Republic; ostadal.petr@gmail.com; 3 Asper Clinical Research Institute, St. Boniface Hospital, Winnipeg, MB R2H 2A6, Canada; ptappia@sbrc.ca

**Keywords:** mitochondria, oxidative stress, Ca^2+^-handling defects, cell death, cardiac dysfunction, heart disease

## Abstract

**Background:** Several mitochondrial abnormalities such as defective energy production, depletion of energy stores, Ca^2+^ accumulation, generation of reactive oxygen species, and impaired intracellular signaling are associated with cardiac dysfunction during the development of different heart diseases. **Methods:** A narrative review was compiled by a search for applicable literature in MEDLINE via PubMed. **Results:** Mitochondria generate ATP through the processes of electron transport and oxidative phosphorylation, which is used as energy for cardiac contractile function. Mitochondria, in fact, are the key subcellular organelle for the regulation of intracellular Ca^2+^ concentration and are considered to serve as a buffer to maintain Ca^2+^ homeostasis in cardiomyocytes. However, during the development of heart disease, the excessive accumulation of intracellular Ca^2+^ results in mitochondria Ca^2+^-overload, which, in turn, impairs mitochondrial energy production and induces cardiac dysfunction. Mitochondria also generate reactive oxygen species (ROS), including superoxide anion radicals and hydroxyl radicals as well as non-radical oxidants such as hydrogen peroxide, which promote lipid peroxidation and the subsequent disturbance of Ca^2+^ homeostasis, cellular damage, and death. **Conclusion:** These observations support the view that both oxidative stress and intracellular Ca^2+^-overload play a critical role in mitochondrial disruption during the pathogenesis of different cardiac pathologies.

## 1. Introduction

Oxidative stress is considered to be an imbalance between reactive oxygen species (ROS) and oxidant production and the state of glutathione redox buffer as well as antioxidant defense systems [1,2]. While ROS, such as superoxide anion radicals and hydroxyl radicals, contribute to oxidative stress, non-radical ROS (oxygen-derived molecules that are not free radicals) that do not have unpaired electrons, such as hydrogen peroxide, singlet oxygen, hypochlorous acid, and peroxynitrite, are also considered highly reactive molecules that contribute to oxidative stress. Although large clinical trials with antioxidant vitamins C and E have not conclusively demonstrated a benefit for cardiovascular diseases (CVDs) [3,4,5], there is clear evidence that most CVDs are linked to or even initiated by oxidative stress [6,7], as indicated by phenotypic changes in animal models of CVDs with genetic deletion or the overexpression of enzymes involved in the synthesis or degradation of ROS [8].

Oxidative stress and intracellular Ca^2+^-overload are intimately involved in different cardiac pathologies, including heart failure, diabetic cardiomyopathy, and ischemia–reperfusion injury [9,10,11,12,13,14,15]. Such defects in cardiomyocyte Ca^2+^-handling have been attributed to subcellular remodeling during the development of heart disease [16,17,18,19,20,21,22]. Mitochondria are the major source of ATP production through oxidative phosphorylation and electron transport systems [23,24,25,26] and are regarded as multifunctional organelles involved in cardiomyocyte function and integrity. Indeed, mitochondria regulate key processes including mitophagy, apoptosis, redox balance, and Ca^2+^ homeostasis [27,28,29,30,31,32]. There is a wealth of information demonstrating that mitochondria are a major source of ROS, which promote lipid peroxidation, leading to a dysregulation of cation homeostasis, cellular damage, and cell death [33,34]. Furthermore, the occurrence of oxidative stress is accompanied by a depletion of antioxidant enzymes and other redox-regulating molecules, which exacerbates the imbalance between ROS generation and detoxification, contributing to the acceleration of myocardial abnormalities in terms of structure and function. It should be mentioned that mitochondria are known to contain different components for the production of ROS, such as the electron transport chain, NADPH oxidase 4, and monoamine oxidase, in addition to endogenous antioxidants such as SOD, CAT, and glutathione peroxidase. Particularly, it may also be noted that the accumulation of different vasoactive hormones such as angiotensin II and endothelin (activators of NADPH oxidase 4), as well as catecholamines and serotonin (substrates for oxidation by MAO), occurs in cardiomyocytes of diseased hearts. Furthermore, the occurrence of mitochondrial Ca^2+^-overload has been associated with the activation of MAO and the induction of defects in electron transport systems in mitochondria in ischemic heart disease.

In view of the importance of mitochondria in normal cell function, this narrative review intends to describe the role of defects in mitochondrial energy generation, increased ROS production, dysregulation of cardiomyocyte Ca^2+^-handling, and mitochondrial Ca^2+^-overload, as well as cell apoptosis in cardiac dysfunction in different pathophysiological conditions such as heart failure, diabetic cardiomyopathy, and ischemia–reperfusion injury. Accordingly, the appropriate literature was searched on MEDLINE via PubMed by using the following search terms: mitochondrial dysfunction, cardiac ischemia–reperfusion injury, diabetic cardiomyopathy, heart failure, reactive oxygen species, oxidative stress, Ca^2+^-handling, and intracellular Ca^2+^-overload, and combinations thereof, and the articles cited in this review were those selected to provide support of our hypothesis.

## 2. Mitochondria as a Source of ROS and Oxidative Stress

**Figure 1:**
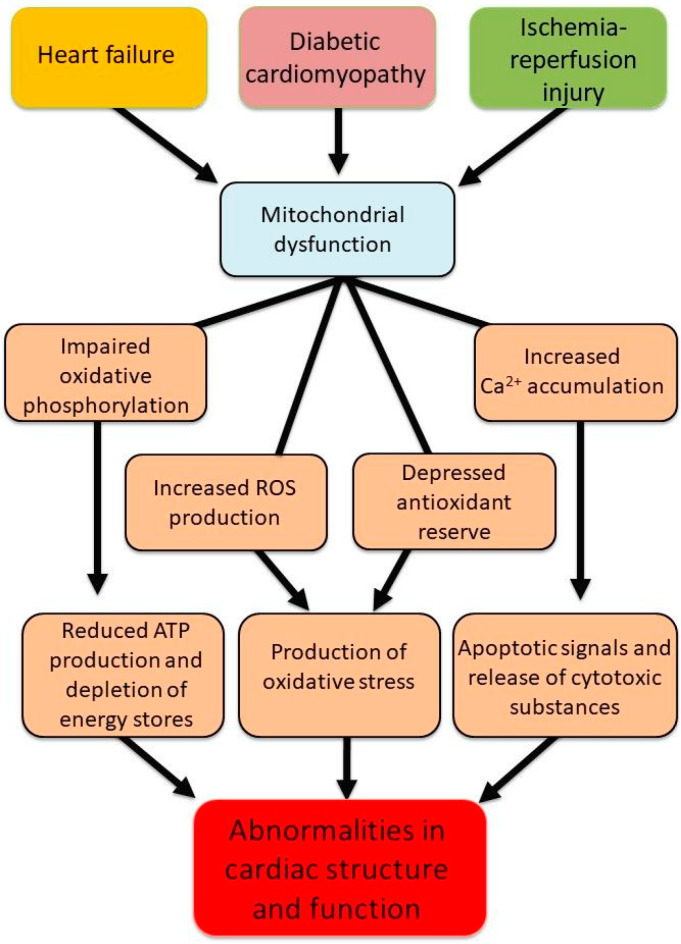
Abnormalities associated with mitochondrial dysfunction leading to changes in cardiomyocyte structure and function in different cardiac pathologies.

**Figure 2:**
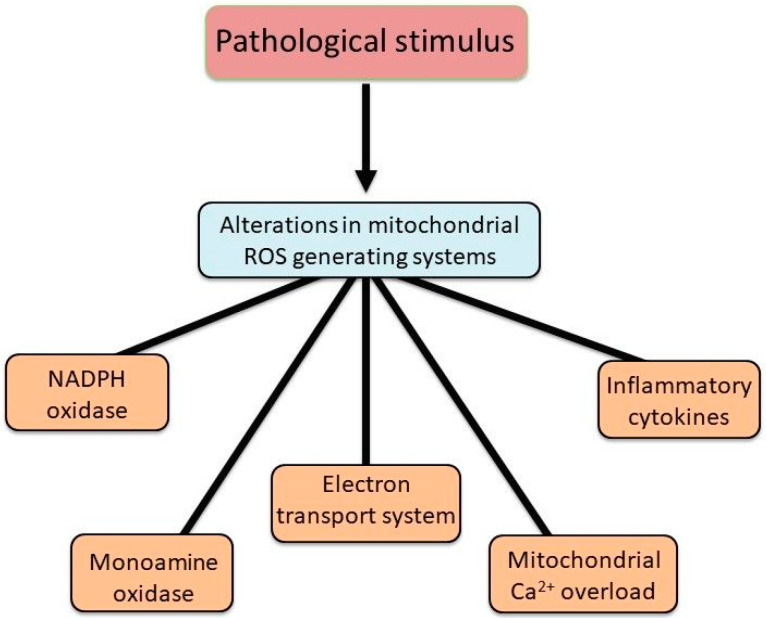
Alterations in different ROS-generating systems in mitochondria due to pathological stimulus. Abbreviation: ROS = reactive oxygen species.

Mitochondria are known to accumulate a considerable amount of Ca^2+^ and are thus considered a Ca^2+^ reservoir/sink designed to maintain the intracellular concentration of free Ca^2+^ ([Ca^2+^]i) within an optimal range [23,24,35]. However, during the development of different cardiac diseases, an excessive amount of cytoplasmic Ca^2+^ results in mitochondria Ca^2+^-overload that subsequently harms mitochondrial energy production [23,24,36]. Taken together, it can be seen from Figure 1 that mitochondrial dysfunction in different types of heart diseases and pathophysiological conditions is a key parameter in the pathogenesis of cardiac dysfunction. This functional decline in the heart is strongly associated with excessive ROS generation, which originates from multiple sources, including NADPH oxidase, monoamine oxidase, and mitochondrial respiratory complexes I, II, and III. Notably, NADPH oxidase 4 (NOX4) has been identified to be present in mitochondria mainly and serves as a principal driver of oxidative stress in heart failure [37]. Figure 2 summarizes the alterations in mitochondrial ROS-generating systems. It should be mentioned that endothelium-associated xanthine oxidase (XO) and NADPH oxidase in endothelial cells, which are known to generate superoxide anion radicals, are activated by angiotensin II [38,39]. This surplus of superoxide anion radicals generated through these pathways induces widespread damage to cellular macromolecules, including DNA, proteins, lipids, and carbohydrates, ultimately resulting in mitochondrial dysfunction and irreversible cytotoxicity. Indeed, the interplay between ROS and mitochondrial components creates a self-amplifying cycle of oxidative damage, further exacerbating mitochondrial dysfunction, contractile impairment, and the overall progression of heart failure [40,41].

**Figure 3:**
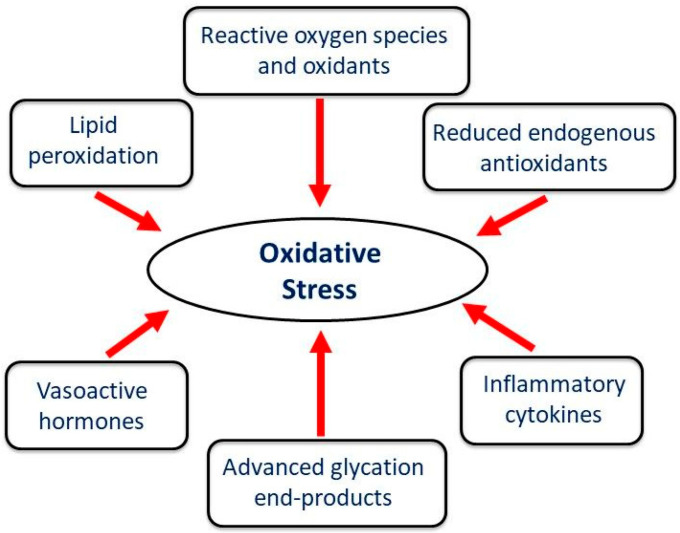
Different factors involved in the development of oxidative stress in diseased heart.

Oxidative stress represents a state of redox disequilibrium characterized by the excessive generation of ROS, including superoxide anion radicals, hydrogen peroxide, and hydroxyl radicals, alongside a concurrent reduction in endogenous antioxidant capacity [42]. It should be mentioned that the hydroxyl radicals are considered the most powerful among the ROS [43]. The accumulation of mitochondrial ROS accompanied by a variety of different factors, including heightened inflammatory response, formation of advanced glycation end-products, and lipid peroxidation, all collectively exacerbate oxidative stress (Figure 3).

The deleterious effects of oxidative stress are not limited to mitochondrial impairment, but they also contribute to the pathological remodeling of the myocardium through the upregulation of pro-inflammatory cytokines and activation of fibroblasts in the extracellular matrix [44,45]. These mechanisms collectively promote interstitial fibrosis and increase myocardial stiffness, which are hallmarks of heart failure progression. It should be mentioned that Nrf2 (nuclear factor erythroid 2-related factor 2), a transcription factor that regulates antioxidant responses, plays a critical role in cellular defense against oxidative stress, inflammation, and apoptosis. It is a key regulator of several genes for endogenous antioxidants such as superoxide dismutase (SOD), catalase (CAT), and glutathione peroxidase, which are involved in protecting against the development of oxidative damage and mitochondrial dysfunction, making it a promising therapeutic target in cardiovascular diseases [46]. In fact, numerous studies suggest that Nrf2 activation is a crucial cardioprotective mechanism against the adverse effects of ischemic myocardial injury.

**Figure 4:**
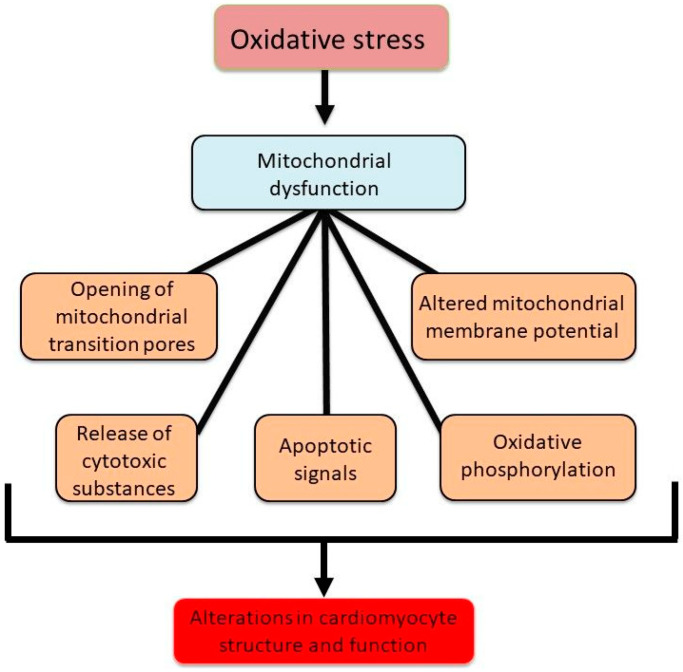
Mitochondrial abnormalities due to oxidative stress, leading to changes in cardiac function and structure.

The prolonged exposure of the heart to high levels of circulating vasoactive hormones including angiotensin II and catecholamines in chronic myocardial infarction has been shown to induce Ca^2+^-handling abnormalities that have been linked to the occurrence of mitochondrial Ca^2+^-overload, mitochondrial dysfunction, and the generation of oxidative stress, all leading to an impairment of cardiovascular function [47,48,49]. Both intracellular Ca^2+^-overload and oxidative stress are considered to induce conformational alterations in the mitochondrial cristae embedded F1/F0, ATP synthase, and permit the formation of membrane permeability transition pores (MPTPs) for releasing solutes and proteins, including cytochrome C, apoptosis-inducing factors, and Smac/DIABLO, from the mitochondrial matrix [50,51,52]. If the MPTPs remain in the open state for some period, cardiomyocytes become unable to sustain their ATP levels, ultimately leading to mitochondrial stress, cell death, and cardiac dysfunction [53]. Taken together, Figure 4 demonstrates the critical role played by mitochondria in alterations in cardiomyocyte structure and function through modulating energy metabolism, formation of the MPTPs, and inducing apoptotic signals.

## 3. Impact of Lipid Peroxidation on Mitochondrial Function

Lipid peroxidation is a destructive process that causes damage to cells and tissues and has been linked to several different pathophysiological conditions, including heart disease [54,55]. In fact, lipid peroxidation products are the major drivers of cell death through necrosis, apoptosis, and ferroptosis. It should be noted that malondialdehyde is generated by the peroxidation of membrane polyunsaturated fatty acids (PUFAs) and is an established biomarker for oxidative stress. Many studies have evaluated the correlation between the levels of lipid peroxidation products and pathological states and their use as biomarkers for the early diagnosis and prognosis of disease [56,57,58]. However, it should be noted that high levels of peroxidized lipids do not necessarily indicate that lipid peroxidation is a cause of the disease, but a positive correlation implies that they can be used as biomarkers [59]. The excess of ROS leads to oxidative stress instigating the peroxidation of PUFAs in the lipid membrane through a free radical chain reaction and the formation of the most bioactive and cytotoxic aldehyde, known as 4-hydroxy-2-nonenal (4-HNE). The excessive production of ROS can lead to an accumulation of 4-HNE inside the mitochondria, which is considered far more harmful than ROS [60]. It functions as a signaling molecule and toxic product and acts mainly by forming covalent adducts with nucleophilic functional groups in proteins, amino acids, and lipids [61,62]. Mitochondria have been implicated as a site for 4-HNE generation and adductions. It has been shown that 4-HNE activates mitochondrial apoptosis-inducing factor (AIFM2) and facilitates apoptosis. In addition, 4-HNE inside mitochondria leads to the adduction of several mitochondrial respiratory chain complex proteins [63].

It has been reported that the amount of 4-HNE, which is a major lipid peroxidation product and a cytotoxic aldehyde, is increased in the human failing myocardium [64]. Furthermore, 4-HNE and hydroxyl radicals in cardiomyocytes play an important role as mediators of oxidative stress in heart failure [64]. 4-HNE rapidly increases [Ca^2+^]i, augments the rate of ROS generation, and causes a loss of mitochondrial membrane potential as well as reduction in ATP and GSH levels; such alterations result in the activation of apoptotic cell death and disruption of the cytoskeleton [65]. Ketoaldehydes, formed by the isoprostane pathway, have been reported to disrupt mitochondrial respiration and Ca^2+^ homeostasis through induction of the MPTPs [66]; indeed, it has been suggested that Ca^2+^ mediates mitochondrial damage through the opening of MPTPs, whereas ROS mediates its effects through lipid peroxidation [67]. It should be mentioned that cardiolipin is a unique phospholipid, which is almost exclusively located at the inner mitochondrial membrane where it is biosynthesized and intimately involved in several mitochondrial bioenergetic processes [68]. Ca^2+^-binding to cardiolipin has been suggested to be an early step in the molecular mechanisms of Ca^2+^-induced nonspecific inner mitochondrial membrane permeabilization [69]. Oxidative damage to cardiolipin would be seen to negatively impact the biochemical function of the mitochondrial membrane, altering membrane fluidity, ion permeability, and the structure and function of the components of the mitochondrial electron transport chain, resulting in reduced mitochondrial oxidative phosphorylation and apoptosis. Interestingly, the intrinsic activity of aldehyde dehydrogenase (ALDH2), a cardiac mitochondrial enzyme, is vital in detoxifying 4-HNE [70] and thus confers cardioprotection against pathological stress [71]. A single mutation (E487K) in ALDH2, that is prevalent in East Asian people, known as ALDH2*2 reduces its activity and increases CVD [71]. On the other hand, SOD2 deficiency (SOD2 knockout mice) increases ROS, leading to the subsequent overproduction of 4-HNE inside mitochondria. Proteins in the mitochondrial respiratory chain complex and in the tricarboxylic acid cycle were reported to be targets of 4-HNE adduction; thus, 4-HNE may be an important factor in heart disease [72]. In fact, 4-HNE decreases mitochondrial oxygen consumption by inhibiting electron transport chain [73].

## 4. Evidence of Involvement of ROS and Ca^2+^-Overload in Cardiac Mitochondria

**Figure 5:**
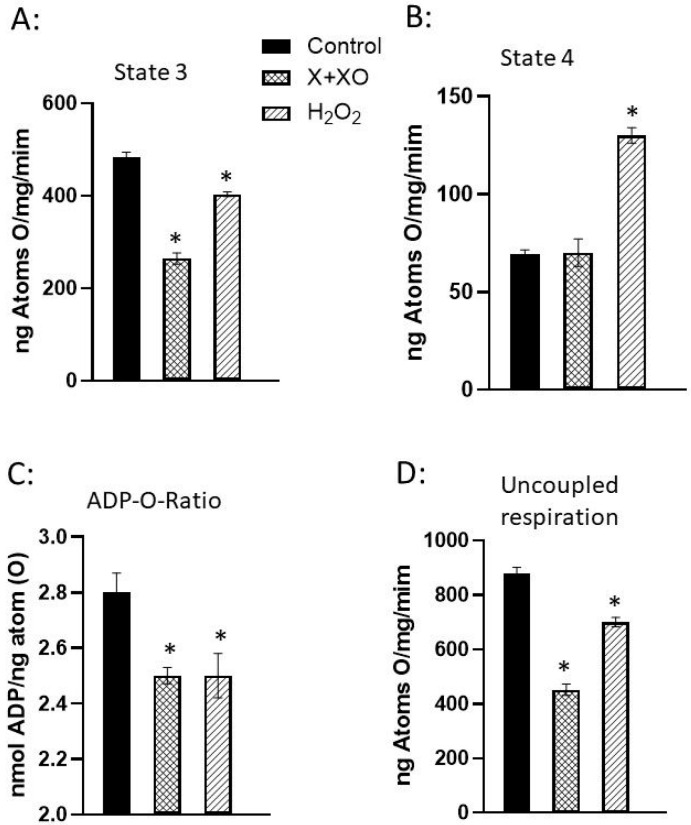
Mitochondrial respiration and oxidative phosphorylation activities of rat hearts perfused with xanthine + xanthine oxidase or H_2_O_2_. Hearts were perfused with 2 mM X and 60 mU/mL XO or with 100 µM H_2_O_2_ for 30 min. (**A**):State 3 respiration; (**B**) State 4 respiration; (**C**) ADP to oxygen ratio and (**D**) Uncoupled respiration. Data are taken from our paper [78]. Values are mean ± SE of 3 experiments. * = *p* < 0.05. Abbreviations: X = xanthine; XO = xanthine oxidase; H_2_O_2_ = hydrogen peroxide.

Mitochondria play a pivotal role in cellular redox signaling by generating ROS as by-products of oxidative phosphorylation [74,75,76]. However, not all oxidants play a role in signal transduction as it appears that this is dependent upon the cell type and animal species. Furthermore, low concentrations of oxidants or exposure for a transient period stimulate the signal transduction mechanisms for both cardiomyocyte function and gene expression for cell survival, while high concentrations of oxidants and/or exposure for a prolonged period of time produce oxidative stress and subsequent harmful outcomes [9,77]. The impairment of mitochondrial function by ROS-generating systems and oxidants has been reported [78,79]. In this regard, normal rat hearts perfused with an ROS-generating system, xanthine (X) plus xanthine oxidase (XO), have been shown to decrease mitochondrial state 3, uncoupled respiration, and the ADP-to-O ratio without any changes in state 4 respiration (Figure 5). On the other hand, perfusion with a well-known oxidant, H_2_O_2_, increased mitochondrial state 4 respiration and decreased the ADP-to-O ratio as well as mitochondrial state 3 and uncoupled respiration (Figure 5) [78]. The role of ROS and oxidants in the mitochondrial impairment of oxidative stress was further demonstrated by the observations that the changes in mitochondrial function due to X plus XO were attenuated or prevented by the presence of SOD plus CAT, whereas those by H_2_O_2_ were attenuated by the presence of CAT plus mannitol, but not by CAT alone (Table 1) [78]. The impact of the oxidant effect on [Ca^2+^]i is demonstrated by the data presented in Table 2. It was observed that the H_2_O_2_-induced increase in [Ca^2+^]i is concentration-dependent (Table 2A) [79]. In contrast, the incubation of cardiomyocytes with CAT before exposure to H_2_O_2_ attenuated the H_2_O_2_-induced increase in [Ca^2+^]i. It should be noted that mannitol did not exert any effect on the H_2_O_2_-induced increase in [Ca^2+^]i (Table 2B) [79]. Taken together, it can be inferred that the formation of H_2_O_2_ in different cardiac pathologies can induce changes in Ca^2+^ homeostasis in cardiomyocytes and induce cardiac contractile dysfunction.

## 5. Development of Mitochondrial Ca^2+^-Overload Due to Oxidative Stress

Mitochondria participate in I/R-injury due to oxidative stress and dysregulation of Ca^2+^ homeostasis [80]. In addition, following I/R, cardiomyocytes accumulate high levels of peroxides, leading to mitochondrial dysfunction and the induction of ferroptosis and exacerbation of ROS production and oxidative stress [81]. The oxidative-stress-induced abnormalities in Ca^2+^-handling are known to lead to mitochondrial Ca^2+^-overload, resulting in the impaired mitochondrial production of energy [82,83,84]. Interestingly, mitochondrial ATPase inhibitory factor-1, which is increased under conditions of oxidative stress, has been reported to disturb mitochondrial Ca^2+^-handling; however, the loss of mitochondrial Ca^2+^ uniporter (mCUP) has been reported to trigger arrhythmias attributed to a probable effect on SR Ca^2+^-handling [85,86]. Interestingly, in I/R-injury, Ca^2+^ influx into mitochondria is considered to occur through the mCUP; however, the deletion of the mCUP has been reported to result in an increase in mitochondrial Ca^2+^, suggesting that some other mechanism may also be involved in Ca^2+^ influx [87]. It should also be mentioned that a defect in the cross talk between mitochondrial function and control of ryanodine-receptor-mediated SR Ca^2+^-release has been linked to an increase in the risk of arrhythmia in heart disease [88]. Clearly, targeting Ca^2+^ homeostasis in cardiomyocytes and mitochondrial Ca^2+^-overload due to oxidative stress would be seen as beneficial in attenuating calcium dysregulation in heart disease, including myocardial infarction, heart failure, and cardiomyopathies [89].

The sensitivity of mitochondria to Ca^2+^ concentrations is critical, as both excessive and deficient Ca^2+^ levels can impair mitochondrial oxidative phosphorylation. High-glucose conditions in cardiomyocytes have been shown to reduce mCUP expression, decrease mitochondrial Ca^2+^ levels, and alter glucose and lipid metabolic profiles, further compromising cardiac function [90]. Mitochondrial Ca^2+^-overload, in turn, contributes to oxidative stress, which exacerbates mitochondrial dysfunction and creates a vicious cycle of cellular injury. This cascade ultimately leads to apoptosis or necrosis, further impairing both systolic and diastolic heart function [91]. In the context of diabetic cardiomyopathy, the role of mCUP and its regulatory subunit, mitochondrial calcium uptake protein 1 (MICU1), has emerged as a critical factor in Ca^2+^-transport. It has been shown that in diabetic mice, there is an upregulation of MICU1 expression in the heart, accompanied by a downregulation of MCU and associated regulatory proteins, such as EMRE, a key mCUP subunit. This imbalance leads to compromised mitochondrial Ca^2+^-uptake, diminished mitochondrial function, and consequently reduced cardiac performance.

Mitochondria Ca^2+^ accumulation serves as a key trigger of mitochondrial dysfunction, especially when it occurs in the presence of additional stressors such as oxidative or nitrosative stress [92]. Ca^2+^ signaling has emerged as a critical modulator of mitochondrial function, with evidence indicating that Ca^2+^ contributes to the initiation of mitochondrion-dependent apoptosis [93]. Mitochondria serve as both ATP producers and crucial intracellular Ca^2+^ buffers. The mCUP, located on the inner mitochondrial membrane, plays a pivotal role in mediating Ca^2+^ influx into the mitochondrial matrix. Under normal physiological conditions, even modest fluctuations in Ca^2+^ levels are sufficient to activate dehydrogenases like FoF1-ATP, promoting ATP synthesis. However, under pathophysiological conditions, these processes are disrupted. Inositol trisphosphate receptors (IP_3_Rs) are essential for maintaining intracellular calcium homeostasis. The release of Ca^2^⁺ from IP_3_Rs functions as a second messenger, orchestrating various intracellular processes and inter-organellar communication in both physiological and pathological contexts. Overactivation of IP_3_Rs has been linked to the pathogenesis of several cardiac disorders, including ischemia, diabetes-induced arrhythmias, and cardiac hypertrophy. Dysregulated Ca²⁺ signaling within cytosolic, mitochondrial, and nucleoplasmic compartments contributes to the progression of these diseases [94]. Interestingly, an adaptive mechanism through which mitochondria mitigate ROS-induced damage is apoptosis induction. This process involves an increase in mitochondrial outer membrane permeability, leading to solute and water influx into the matrix, loss of membrane potential, cessation of ATP synthesis, and excessive mitochondrial calcium uptake, culminating in complete mitochondrial failure [95].

## 6. Mitochondrial Metabolic Alterations and Mitochondrial Dynamics

It is well established that mitochondrial dysfunction is a hallmark of cardiovascular diseases (CVDs), manifesting as impaired oxidative phosphorylation, excessive ROS production, altered calcium signaling, and disrupted metabolic homeostasis. Conditions such as ischemia–reperfusion injury, hypertension, and diabetic cardiomyopathy are associated with compromised mitochondrial energetics and structural integrity, culminating in cardiac contractile dysfunction [96,97,98,99,100,101,102]. The heart is an energetically demanding organ, necessitating a continuous and substantial supply of adenosine triphosphate (ATP) to sustain contractile function. Despite its limited ATP reserves, the heart maintains an exceptionally efficient bioenergetic system, predominantly driven by mitochondria, which constitute nearly 30% of cardiomyocyte volume. These organelles facilitate ATP generation through oxidative phosphorylation, orchestrating substrate oxidation, electron transport, and ATP synthesis to meet the heart’s metabolic demands [103,104,105,106,107]. Mitochondrial function is intricately linked to substrate availability, with ATP synthesis reliant on the oxidation of fatty acids, glucose, ketone bodies, and amino acids. It should be mentioned that biologically active amines such as spermine and agmatine play distinct roles in mitochondrial function that differentiate them from other amines; notably, spermine undergoes oxidative deamination by amine oxidases, producing ROS, which may further exacerbate the opening of the MPTPs and contribute to apoptosis [52].

**Figure 6:**
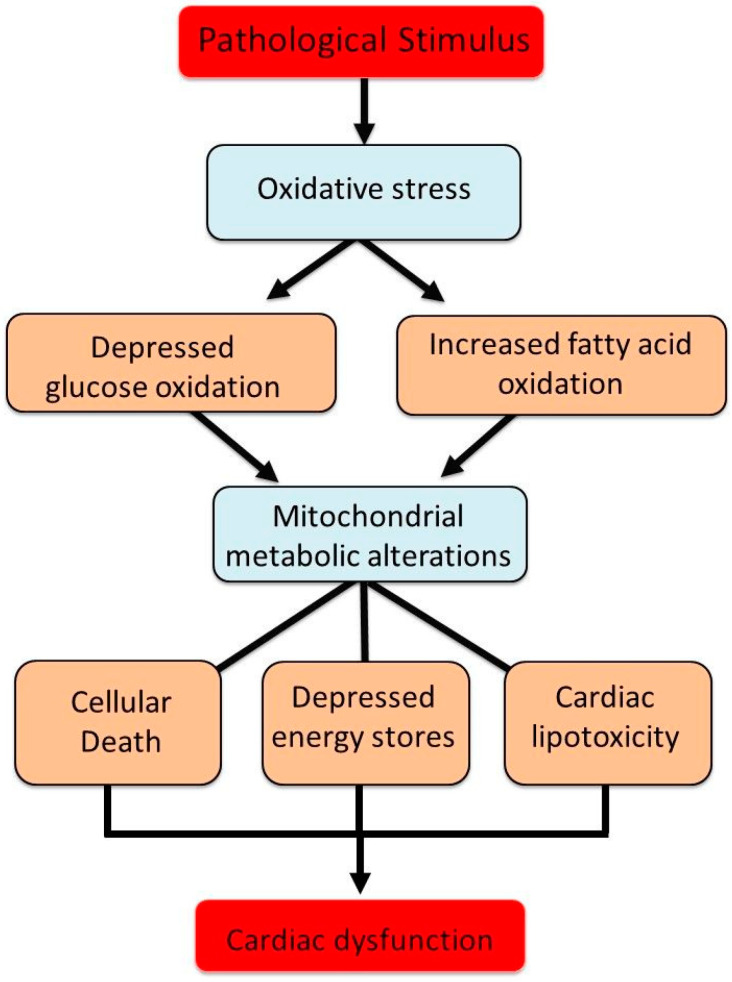
Involvement of oxidative stress in inducing changes in mitochondrial metabolism for the occurrence of cardiac dysfunction due to pathological stimulus.

In the healthy myocardium, fatty acid oxidation contributes approximately 60–70% of ATP production, while glucose metabolism accounts for 20–30% [108,109,110,111]. The efficiency of ATP synthesis per unit of oxygen is higher for glucose than for fatty acids, a critical factor under hypoxic or ischemic conditions [112,113,114,115]. The intrinsic compensatory mechanisms that regulate intracellular calcium and the antioxidant defense system, which typically maintain mitochondrial substrate oxidation and ATP generation, become insufficient in the context of chronic cardiac dysfunction [116,117,118]. It should be mentioned that insulin resistance in diabetes reduces glucose transporter expression and pyruvate dehydrogenase activity, shifting myocardial energy reliance towards fatty acid β-oxidation. This metabolic shift increases oxygen consumption while decreasing ATP yield efficiency, predisposing mitochondria to oxidative stress and lipotoxic damage [119,120,121,122]. Prolonged metabolic perturbations, including excessive fatty acid uptake and β-oxidation inefficiencies, promote lipid accumulation, mitochondrial dysfunction, and cardiomyocyte apoptosis. These maladaptive changes contribute to myocardial energy deficits, compromised contractility, and heightened susceptibility to heart failure [123,124,125,126,127,128]. In addition, the chronic dysregulation of glycolipid metabolism in diabetes leads to both excessive ROS production and impaired ROS clearance. Mitochondria serve as the primary source of ROS in diabetic cardiomyocytes, and their dysfunction perpetuates a vicious cycle of oxidative damage. This process severely compromises cardiomyocyte function and survival by exacerbating metabolic disturbances, energy depletion, and oxidative stress-driven apoptosis [129]. However, it should be noted that chronic hyperglycemia during diabetes further increases mitochondrial ROS production and impairs endogenous antioxidant defense mechanisms, thus leading to excessive apoptosis and myocardial dysfunction [130,131]. A schematic diagram indicating the role of oxidative stress in inducing mitochondrial metabolic changes associated with depression in energy stores, cellular death and lipid deposits in cardiomyocytes, and subsequent cardiac dysfunction in diseased hearts is shown in Figure 6.

It should be pointed out that mitochondrial-generated oxidative stress, signal transduction, metabolic reprogramming, and the regulation of iron and cell death depend on the mitochondrial quality control (MQC) system, which includes mitochondrial dynamics (fission and fusion cycles) and mitochondrial biogenesis to maintain structural integrity and cardiac function [132,133,134]. Thus, targeting mitochondrial bioenergetics and metabolic flexibility represents a promising therapeutic strategy for mitigating CVD progression and preserving cardiac function. The mitochondrial ribosomal protein S5 (MRPS5/uS5m) is essential for maintaining mitochondrial protein translation and oxidative phosphorylation. Loss of MRPS5 in the developing heart leads to embryonic lethality, while postnatal loss impairs oxidative phosphorylation and mitochondrial protein synthesis, contributing to cardiac hypertrophy and heart failure [135]. Mitochondrial dynamics, including fusion and fission processes, are integral to maintaining mitochondrial function and integrity [136]. Fusion proteins such as Mfn-1, Mfn-2, and OPA-1 are essential for mitochondrial stability, as their inhibition leads to dilated cardiomyopathy, contractile dysfunction, increased apoptosis, and mitochondrial fragmentation [137]. The deletion of these fusion proteins results in abnormal mitochondrial morphology, ventricular wall thickening, and eccentric hypertrophy. Conversely, excessive mitochondrial fission disrupts mitochondrial mass, impairs oxidative phosphorylation, and results in ATP deficits, mitochondrial permeabilization, cytochrome C release, and apoptosis. The absence of dynamin-related protein 1 (Drp1), a key fission protein, results in lethal dilated cardiomyopathy [138], further highlighting the critical balance between fusion and fission in maintaining cardiac mitochondrial function.

Alterations in mitochondrial ultrastructure and bioenergetics are widely observed in heart failure patients, particularly the later disease stages. These changes include reductions in the activities of respiratory chain complexes (I–IV) and impaired oxidative phosphorylation capacity. Interestingly, in cases of chronic hypertrophy without systolic dysfunction, mitochondrial function appears to be preserved or even enhanced in both animal models and human studies [139,140,141,142,143,144,145,146,147,148,149,150]. Initial stages of cardiac hypertrophy are often characterized by an increase in oxidative phosphorylation activity, which gradually declines as the disease progresses toward heart failure [24,151]. A reduction in the expression of key oxidative phosphorylation components has been linked to mitochondrial respiratory deficits in heart failure and cardiomyopathies [152,153]. It is pointed out that in ventricular fibrillation, mitochondrial damage activates the mitochondrial apoptotic pathway, characterized by the release of cytochrome C into the cytosol, a reduction in caspase-9 levels, and the subsequent activation of caspase-3. This cascade coincides with significant impairments in LV function. Notably, cytochrome C “leaks” into the bloodstream, and its concentration is inversely proportional to survival outcomes [154]. Taken together, these findings underline the critical role of mitochondria during cardiac resuscitation by modulating both energy metabolism and apoptotic signaling pathways, positioning mitochondrial-targeted therapies as promising strategies for enhancing outcomes during cardiac resuscitation [126].

## 7. Novel Interventions Targeting Mitochondria in Different Cardiac Pathologies

In view of the fact that mitochondria constitute 30–40% of the cardiomyocyte [155], and that mitochondria are considered the key determinants of cardiac injury and dysfunction [156,157,158,159,160], mitochondria represent a viable target for therapeutic intervention. Indeed, mitochondrial dysfunction is characterized by impaired bioenergetics, oxidative stress, and aldehydic load and is considered a hallmark of heart failure. The selective activation of mitochondrial detoxifying systems that counteract the excessive accumulation of ROS and reactive aldehydes is emerging as an adequate tool to inhibit cardiac degeneration in heart failure [55]. Thus, pharmacological and non-pharmacological approaches targeting mitochondria detoxification could play a key role in the prevention or treatment of heart failure. For example, the regulation of mitochondrial Ca^2+^-uptake and the interplay between various molecules and pathways offer promising avenues for therapeutic intervention. Mitochondria have been suggested to exhibit a cardioprotective role due to the presence of K_ATP_ channels [161]. In this regard, cardioprotection via hypoxic preconditioning or exposure to the ATP-dependent K^+^-channel opener, diazoxide, increases mitochondrial resistance to oxidative damage. Thus, targeting the MPTPs, either by direct inhibition or modulation of mitochondrial stressors, represents a promising therapeutic approach for conditions such as I/R-injury [93,162]. Interestingly, the reperfusion injury salvage kinase (RISK) pathway for cardioprotection involves the prevention of the opening of the membrane permeability transition pore and subsequent attenuation of cell death [163]. Thus, this pathway has emerged as an important cardioprotective target in I/R-injury.

Reperfusion-induced injury is a significant challenge during cardiac surgery, coronary thrombosis treatment, and stroke management. Preventing MPTP opening, either directly with agents like cyclosporine A or indirectly by reducing oxidative stress or Ca^2+^-overload, represents a potential therapeutic strategy to mitigate reperfusion injury. Additionally, mice deficient in Cyclophilin D (CyP-D), a critical component of the MPTPs, are protected from ischemia–reperfusion injury in the heart, further substantiating the role of MPTPs in mediating cellular injury [164]. In less severe cellular insults, the MPTPs may open transiently, leading to mitochondrial swelling sufficient to trigger cytochrome C release and activation of the apoptotic pathway, rather than necrosis. However, CyP-D knockout mice do not exhibit enhanced protection against a broad range of apoptotic stimuli, suggesting that the MPTP is not universally involved in apoptosis [164]. Recently, it has been suggested that circadian rhythm may play an important role in the control of I/R-injury [165]. The molecular application of exosomes has also been explored as potential therapeutic agents in MI; indeed, beneficial effects on heart function and attenuation of ventricular remodeling have been reported, thereby providing support for the clinical application of exosomes in myocardial ischemic injury [166].

MiRNAs regulate mitochondrial apoptosis through an effect on mitochondrial fission and fusion, generation of ROS, and dysregulating Ca^2+^ homeostasis [167]. Interestingly, it has been suggested that mitochondrial transplantation has the potential to exert beneficial actions in I/R-injury; however, clinical application is limited [168]. On the other hand, the potential therapeutic role of miRNAs, specifically mitochondria regulatory genes controlled by miR-15a, miR-29a, and miR-214, may exhibit a therapeutic role in valvular heart disease [169]. Mitochondrial regeneration can be seen to increase oxidative phosphorylation and decrease oxidative stress and thus may be of clinical value under conditions of ischemic insult of the heart [170]. In fact, targeting MQC has emerged as a promising target in mitigating hypoxia-related cardiac dysfunction [171]. Interestingly, it has been proposed that therapeutic approaches to preserve the morphology and function of mitochondria could serve as an important tool in the strategy for cardioprotection [172].

In the context of diabetic cardiomyopathy, the role of mCUP and its regulatory subunit, mitochondrial calcium uptake protein 1 (MICU1), has emerged as a critical factor in Ca^2+^-transport. Studies have shown that in diabetic mice, there is an upregulation of MICU1 expression in the heart, accompanied by a downregulation of MCU and associated regulatory proteins, such as EMRE, a key mCUP subunit. This imbalance leads to compromised mitochondrial Ca^2+^-uptake, diminished mitochondrial function, and, consequently, reduced cardiac performance. Importantly, restoring MCU expression has been shown to ameliorate both mitochondrial and cardiac dysfunction, highlighting the therapeutic potential of restoring mitochondrial Ca^2+^ homeostasis in diabetic cardiomyopathy treatment [173,174]. The expression pattern of the MCU complex subunits MCU and MICU1 has been reported to be markedly increased in aortic valve stenosis and thus besides modifications of cytosolic calcium handling, impaired mitochondrial Ca^2+^-uptake might be an important factor in the progression of septal hypertrophy caused by aortic valve stenosis disease [175]. Distinct isoforms of IP_3_Rs, IP_3_R1, and IP_3_R2 exhibit different roles in cardiac pathology. IP_3_R1 is particularly involved in cardiac ischemia and arrhythmias associated with diabetes, while IP_3_R2 is implicated in sepsis-induced cardiomyopathy and hypertrophy [94]. Thus, IP_3_Rs have been shown to play pivotal roles in various forms of cell death, such as apoptosis, pyroptosis, and ferroptosis, underlining their multifaceted involvement in cardiac disease. Targeting IP_3_Rs, either through genetic manipulation or pharmacological inhibition using IP_3_R antagonists, has emerged as a promising therapeutic strategy to mitigate IP_3_R-related pathologies, offering potential for therapeutic intervention in CVD [94].

A number of studies have examined the clinico-pathological correlations between molecular alterations of mitochondria/cardiomyocytes in the pathophysiology of various cardiac diseases that may have the potential to act as molecular targets for mitochondrial dysfunction and different cardiac pathologies. For example, in patients with hypertrophic cardiomyopathy, a pathogenic mutation of mitochondrial DNA has been reported to lead to mitochondrial dysfunction [176]. It was suggested that the m.4395A>G variant may exert a negative effect on heart function. Mitochondrial proteome studies have also shown that variations in mitochondrial protein expression may play a critical role in the development of human dilated cardiomyopathy [177]. Similarly, in arrhythmogenic cardiomyopathy, transcriptome analysis has revealed 327 genes that were more expressed and 202 genes that were less expressed in arrhythmogenic cardiomyopathy [178]. In this regard, the genes involved in mitochondrial respiration were more expressed, and functional analysis revealed that more active mitochondria and ROS production were in evidence. Taken together, it was demonstrated that the molecular pathways involved in the pathogenesis of arrhythmogenic cardiomyopathy could constitute novel targets [178]. It has been reported that patients with idiopathic dilated cardiomyopathy that do not respond to standard treatments (non-responders) have a reduced expression level of the mitochondrial fusion protein mitofusin-1. Thus, therapies that target mitochondrial dynamics and homeostasis could be of importance in patients with non-responding heart failure [179]. The MT-TL1 gene codes for the mitochondrial leucine transfer RNA that is required for mitochondrial translation. The m.3250T>c variant in the MT-TL1 gene was linked to hypertrophic cardiomyopathy and had an effect on mitochondrial respiration [180].

In human atrial cardiomyocytes, PITX2 deficiency (a paired-like homeodomain transcription factor 2) leads to atrial mitochondrial dysfunction and a metabolic shift to glycolysis [181]. The role of mitochondrial ribosomal protein, L7L12 (MRPL12), in patients with diabetic ischemic heart disease has been examined in right atrial appendage tissues from patients with diabetes undergoing coronary bypass graft surgery [182]. In this examination, an increase in MRPL12 levels in heart samples from diabetic patients with ischemic heart disease was observed, and it was suggested that this increase may be associated with the impairment of mitochondrial membrane potential and alterations in respiration oxygen consumption that could be involved in the pathogenesis of MI in diabetes [182]. It is interesting to note that patients with primary mitral regurgitation hearts showed alterations in metabolic gene profile consistent with a reduction in fatty acid as well as glucose metabolism that were linked to mitochondrial damage despite normal LV function [183]. Cardiac defects and early death, due to variants in the CRLS1 gene that code for cardiolipin biosynthesis, have been reported in individuals with autosomal recessive multisystem mitochondrial disease [184]. In addition, while a lower myocardial cardiolipin content in patients with single right ventricle congenital heart disease has been observed, the expression of genes involved in cardiolipin biosynthesis was upregulated, which may be compensatory in nature. Despite these findings, cardiolipin could serve as a novel therapeutic target in patients with single right ventricle congenital heart disease [185]. These observations regarding the status of mitochondrial function in different CVDs appear to suggest that mitochondrial dysfunction may play a causative role in the pathogenesis of cardiac dysfunction. This view is also supported by the fact that various interventions, which are protective of mitochondrial function, exert beneficial effects in various cardiovascular pathophysiological conditions.

## 8. Conclusions

While the pathophysiology of heart disease is complex and multifactorial, it may be suggested, in view of the foregoing discussion, that mitochondrial dysfunction is a major cause of cardiac disorder. It is evident that mitochondria are not only involved in energy production but are also the major source of oxidative stress production as well as intracellular Ca^2+^ accumulation. In fact, the development of oxidative stress and the occurrence of mitochondrial Ca^2+^-overload are the main mechanisms for the induction of energy store depletion and cardiac dysfunction. Particularly, the impairment of the electron transport system and the activation of mitochondrial NADPH oxidase 4 are the main contributors to ROS formation. It is noteworthy that ROS generated by the activation of sarcolemmal NADPH oxidase 2 as well as extra-mitochondrial (endothelial cells, serum, cytosol) xanthine oxidase are also considered to promote the generation of mitochondrial oxidative stress during the development of heart disease [15,16,186,187,188,189]. There is now a wealth of information that has demonstrated that mitochondrial Ca^2+^-overload and increased generation of ROS are central features in cardiac dysfunction in different cardiac pathologies, including heart failure, diabetic cardiomyopathy, and ischemia–reperfusion injury. Although mitochondria accumulate high amounts of Ca^2+^ and thus serve as an intracellular Ca^2+^ reservoir, abnormalities in the processes involved in energy production through oxidative phosphorylation produce an oxidative stress that impacts the structural and functional integrity of the cell. Indeed, ROS-induced ROS production by mitochondria exacerbates ROS generation and the severity of oxidative stress. The mitochondria-generated ROS as a consequence of mitochondrial Ca^2+^-overload lead to the further deterioration of mitochondrial function. Accordingly, mitochondria present a viable therapeutic target for the prevention of cardiac dysfunction in at-risk populations. Therefore, the development of specific interventions that are effective in attenuating mitochondrial metabolic alterations as well as the development of novel antioxidants that target mitochondrial ROS-generating systems could be highly beneficial.

## Figures and Tables

**Table 1:** Modification of ROS-induced mitochondrial oxidative phosphorylation by antioxidants.

	ADP-to-O Ratio (nmol ADP/ng Atom O)	Uncoupled Respiration (ng Atoms O/min/mg Protein)
A. X + XO Effects		
Control	3.0.6 ± 0.15	575 ± 9
X + XO	2.55 ± 0.07 *	196 ± 7 *
X + XO + SOD + CAT	2.81 ± 2.0.04 #	426 ± 30 *#
B. H_2_O_2_ Effects		
Control	3.13 ± 0.09	543 ± 29
H_2_O_2_	2.37 ± 0.03 *	153 ± 5 *
H_2_O_2_ + CAT	2.52 ± 0.04 *	170 ± 7 *
H_2_O_2_ + CAT + MAN	2.84 ± 0.11 #	195 ± 12 *#

Mitochondria isolated from unperfused hearts were incubated with 0.3 mM xanthine (X) and 11 mU xanthine oxidase (XO) for 3 min at 37 °C. For antioxidant treatment, mitochondria were exposed for 2 min in the presence of 50 U/mL SOD and 50 U/mL CAT before being exposed to X plus XO for 2 min. To study the effects of H_2_O_2_, mitochondria were incubated with a 30 µM concentration of H_2_O_2_ for 3 min. The effect of CAT (8 mU/mL) or mannitol (20 mM) was examined by the pretreatment of mitochondria for 2 min before exposure to 20 µM H_2_O_2_ for 3 min. All these preparations were washed twice and resuspended in a buffer to measure respiratory activities. * *p* < 0.05 vs. control; # *p* < 0.05 vs. respective value in the presence of X + XO or H_2_O_2_ alone. Values are means ± SE of 8 experiments. Data are from our paper (Makazan et al.) [78].

**Table 2:** Modification of H_2_O_2_-induced increase in intracellular Ca^2+^ concentration by antioxidants.

	Increase in [Ca^2+^]i in Cardiomyocytes (% of Control)
A. H_2_O_2_-induced [Ca^2+^]i	
Control	100
0.25 mM	141 ± 11 *
0.5 mM	168 ± 17 *
0.75 mM	216 ± 12 *
1.0 mM	240 ± 23 *
B. Antioxidants on H_2_O_2_-induced [Ca^2+^]i	
Control	52.8 ± 4.7
CAT	14.6 ± 2.0 *
MAN	48.9 ± 5.6
CAT + MAN	8.7 ± 2.5 *

Concentration-dependent effects of H_2_O_2_ on rat cardiomyocyte [Ca^2+^]i. Data shown in (A) were recorded 10 min after incubation of Fura-2-loaded cells (10~/mL) with different concentrations of H_2_O_2_. Fura-2-loaded cardiomyocytes (10~ cells/mL) were treated with 10 µg/mL catalase (CAT), 20 mM mannitol (MAN), or both and a blank buffer (control) for 10 min before exposure to 0.5 mM H_2_O_2_. The concentration of Ca^2+^ in the incubation medium was 1 mM. Fluorescent signals were recorded 10 min after the addition of H_2_O_2_ (B). Control value for [Ca^2+^]i is 120.9 ± 8.1 mM. Data are expressed as means ± SEM of 6–8 experiments. * *p* < 0.05 vs. control. The data are taken from our paper (Wang et al.) [79].

## Data Availability

The original contributions presented in this study are included in the article.
